# Hybrid de novo genome-reassembly reveals new insights on pathways and pathogenicity determinants in rice blast pathogen *Magnaporthe oryzae* RMg_Dl

**DOI:** 10.1038/s41598-021-01980-2

**Published:** 2021-11-25

**Authors:** Bhaskar Reddy, Aundy Kumar, Sahil Mehta, Neelam Sheoran, Viswanathan Chinnusamy, Ganesan Prakash

**Affiliations:** 1 Division of Plant Pathology, ICAR-Indian Agricultural Research Institute, New Delhi, 110012 India; 2 Crop Improvement Group, International Centre for Genetic Engineering and Biotechnology, New Delhi, 110067 India; 3 Division of Plant Physiology, ICAR-Indian Agricultural Research Institute, New Delhi, 110012 India

**Keywords:** Comparative genomics, Genomics, Next-generation sequencing

## Abstract

Blast disease incited by *Magnaporthe oryzae* is a major threat to sustain rice production in all rice growing nations. The pathogen is widely distributed in all rice paddies and displays rapid aerial transmissions, and seed-borne latent infection. In order to understand the genetic variability, host specificity, and molecular basis of the pathogenicity-associated traits, the whole genome of rice infecting *Magnaporthe oryzae* (Strain RMg_Dl) was sequenced using the Illumina and PacBio (RSII compatible) platforms. The high-throughput hybrid assembly of short and long reads resulted in a total of 375 scaffolds with a genome size of 42.43 Mb. Furthermore, comparative genome analysis revealed 99% average nucleotide identity (ANI) with other oryzae genomes and 83% against *M*. grisea, and 73% against *M. poe* genomes. The gene calling identified 10,553 genes with 10,539 protein-coding sequences. Among the detected transposable elements, the LTR/Gypsy and Type LINE showed high occurrence. The InterProScan of predicted protein sequences revealed that 97% protein family (PFAM), 98% superfamily, and 95% CDD were shared among RMg_Dl and reference 70-15 genome, respectively. Additionally, 550 CAZymes with high GH family content/distribution and cell wall degrading enzymes (CWDE) such endoglucanase, beta-glucosidase, and pectate lyase were also deciphered in RMg_Dl. The prevalence of virulence factors determination revealed that 51 different VFs were found in the genome. The biochemical pathway such as starch and sucrose metabolism, mTOR signaling, cAMP signaling, MAPK signaling pathways related genes were identified in the genome. The 49,065 SNPs, 3267 insertions and 3611 deletions were detected, and majority of these varinats were located on downstream and upstream region. Taken together, the generated information will be useful to develop a specific marker for diagnosis, pathogen surveillance and tracking, molecular taxonomy, and species delineation which ultimately leads to device improved management strategies for blast disease.

## Introduction

Since the traditional times, both cereal and cereal products act as pre-eminent and substantial carbohydrate food resources for much of the human population, especially in Asian countries^1,2^. Among the pre-harvest production constraints, the global cultivation of many cereals including rice and pearl millet is mostly affected by a blast disease-causing filamentous ascomycete fungus, *Magnaporthe* (Hebert) Barr (anamorph: *Pyricularia*)^3^. This devastating hemibiotrophic pathogen belongs to the family *Magnaporthaceae* and is of principal concern due to the wide distribution, rapid aerial transmissions, seed-borne latent infection, and associated yield losses^2,4,5^. Morphologically, it causes white, bluish, or greyish water-soaked lesions in all foliar parts such as grain, leaf, neck, collar, nodes, and even panicles^4^. The fungus *Magnaporthe* sp. propagates via the generation of asexual spores (conidia) that disseminate the blast disease on cereal hosts^6,7^.

Out of all reported species belonging to the genus *Magnaporthe*, both *Magnaporthe oryzae* (Anamorph: *Pyricularia oryzae*) and *Magnaporthe grisea* (Anamorph: *Pyricularia grisea*) have emerged as forage and grain production affecting pathogen that annually contributes to the substantial losses of grains^3,4,8^. In order to control the spread and minimize the associated losses with the blast, farmers depend largely on resistant host cultivars, and application of fungicides especially tricyclazole^9^. However, these measures seem inadequate, and inefficient in blast-endemic regions because of related cost, unstable host resistance, the emergence of new pathotypes, toxic nature of the employed agrochemicals, and chemical residues on produced grains. Hence, the global sustainability of food is becoming a more challenging factor regarding the fast growth of the global population^1,4,10^.

Due to the recent advancements in molecular techniques, genes expressed by pathogens (*M. oryzae* and *M. grisea*) for the efficient invasion, colonization, and ultimate destruction of various monocot hosts especially the rice have been characterized^10^. Given that both pathogenic species uniquely attack their host plants, in depth insights into underlying biological pathways and associated mechanisms are still limited with spatial concern to signaling pathways, virulence factors, and carbohydrate-active enzymes. Moreover, the detected genes or proteins can be utilized as potential targets for marker development to screening the pathogen and fungicides through in silico approach^11,12^.

There is a wide range of pieces of literature available on fungal pathogen function and their pathogenesis. However, detailed comparative insights of tropical habitat rice blast fungus genes, family and biochemical pathways are still limited/unexplored. Therefore, to characterize such differences at the genomic and biochemical level, we performed the high-throughput whole-genome sequencing of cereal crop rice blast pathogen *M. oryzae* RMg_Dl followed by hybrid de novo genome assembly and comparative functional annotation. The genome-level analysis potentially deciphers the insights of each genes, their associated biochemical pathways; network for host–pathogen interaction; growth, evolutionary relationship, and virulence genes. The detailed insights of *M. oryzae* not only about host–pathogen interaction but also in-depth knowledge of pathogenicity mechanisms can be helpful for the effective disease management strategy.

## Materials and methods

### Data collection and sequencing

**Figure 1:**
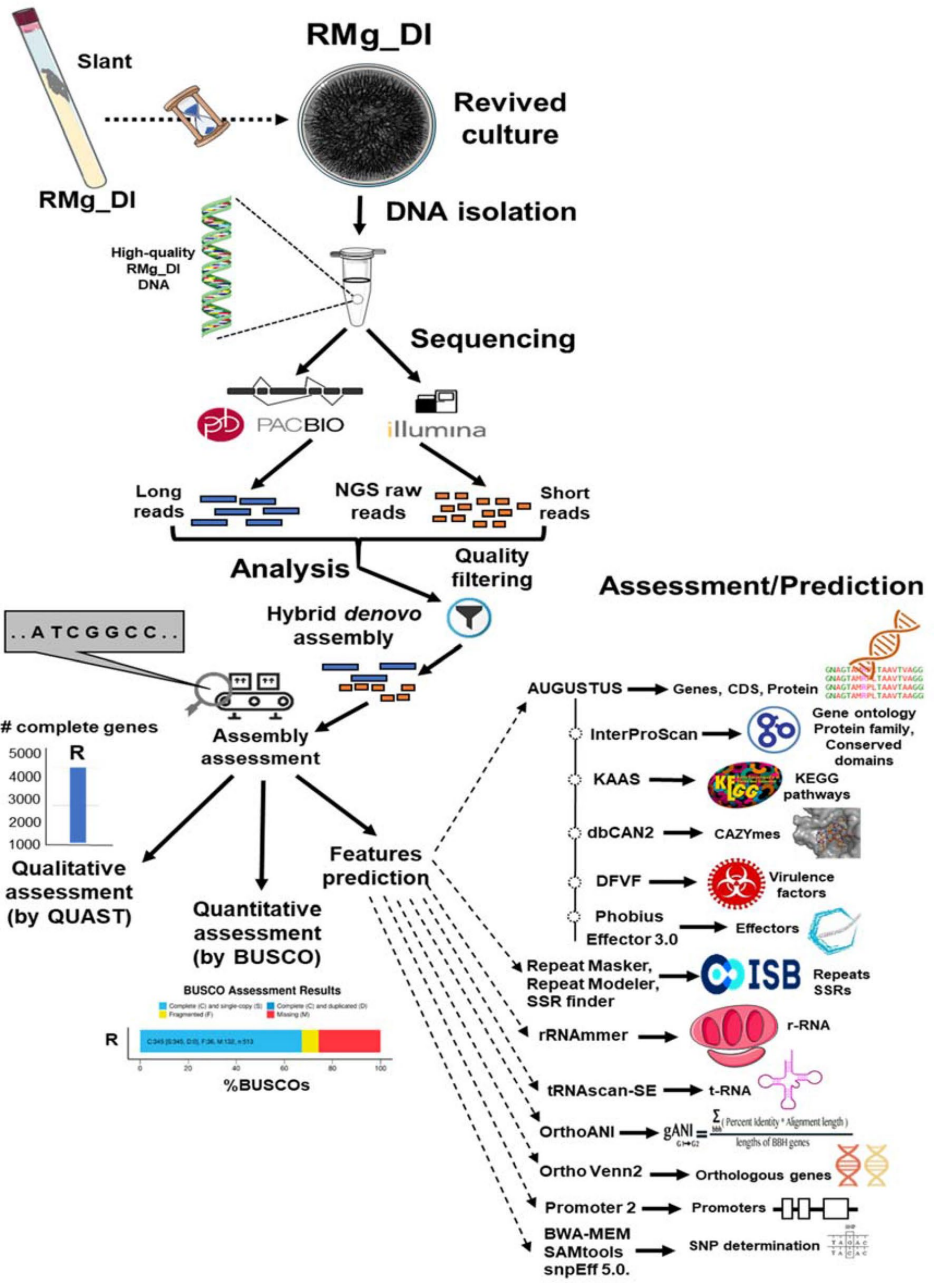
Schematic illustration of the WGS analysis of *M. oryzae* RMg_Dl.

In this study, the used data sets were collected from^13^ (Bioproject accession PRJNA330763, https://www.ncbi.nlm.nih.gov/bioproject/?term=PRJNA330763), which reported the initial draft genome assembly of rice blast disease-causing *Magnaporthe oryzae* RMg_Dl. The sample processing and whole-genome analyses workflow is schematically depicted in Fig. 1. In summary, the pure fungus RMg_Dl was﻿ isolated followed by genomic DNA extraction, and subsequently, the quality and quantity assessment was done using agarose gel (0.8%) and Nanodrop spectrophotometer, respectively. The pair-end library was prepared was using Illumina TruSeq Nano DNA HT chemistry, and we also prepared the single-molecule real-time (SMRT) libraries compatible for generating long reads. The quantity and quality of both libraries were checked on Agilent Bioanalyzer using high sensitivity DNAchip. The Illumina platform quality passed libraries were sequenced on the HiSeq2500 sequencer with 125 × 2 pair-end chemistry. The SMRT-bell libraries were sequenced on PacBio-RSII using P6-C4 chemistry as described here^13,14^.

### Bioinformatic genome assembly and quality assessment

Initially, the reads were visualized in FastQC (V0.9.2) software to screen the quality of reads and identify the poor-quality reads for getting optimum read trimming and filtering parameters. Following this, the WGS reads were quality filtered to remove the adapter, poor sequences, and ambiguous bases to obtain high quality reads with filtering parameters such as reads with unknown nucleotides “N” larger than 5%, low-quality sequences (reads with more than 10% quality threshold (QV) < 20 Phred score) and reads shorter than 100 bases were trimmed using Trimmomatic v0.35^15^. The RMg_Dl genome reads were assembled with a default setting using steps (1) PacBio read correction with LORDeC^16^ using Illumina reads, (2) corrected reads assembly with wtdbg2 assembler^17^, (3) gaps were filled with Long Read Gap closer^18^. Following genome assembly, the benchmark universal single-copy orthologs (BUSCO) was applied for the quantitative assessment of genome completeness against fungal lineage with gene model parameters^19^. Further, to determine the average nucleotide identity (ANI) between publically available genomes and generation of unweighted pair group method with arithmetic mean (UPGMA) tree, the OrthoANI calculator was utilized with default settings^20^.

### Functional genome annotation of *M. oryzae* RMg_Dl

The functional annotation of assembled genome RMg_Dl was performed using the GenSAS web server^21^ which provided an integrated structured pipeline for repeat masking, ab initio gene prediction, homology-based gene function determination, protein family, and superfamily. Initially, the repeat masking was performed using Repeat Masker v4.0.7^22^ against the fungi library with keeping GC content set at 50–52%. Further, Repeat Modeler v1.0.11 (http://www.repeatmasker.org/) was utilized for de novo repeat family identification and modeling. The assembled genome was submitted to AUGUSTUS v3.3.1^23^ for genes and proteins prediction against the reference model *Pyricularia grisea* with both strand gene settings. The prediction of rRNA and tRNA was performed using RNAmmer v1.2^24^ and tRNAscan-SE v2.0^25^. The determination of simple sequence repeats (SSR) was performed with SSR Finder v1.0^21^ with parameters of 5 count repeat for di, 4 for a tri, and 3 for tetra, Penta, and hexanucleotide SSR repeats.

The InterProScan (version5.48-83.0)^26^ of predicted protein sequences were performed to determine the occurrence of various protein families, conserved domains, superfamily, and gene ontology (GO) identifiers in the genome. Following, Bast2GO v5.2.5^27^ was used for the mapping and annotations of GO identifiers to GO terms. The comparative carbohydrate-active enzymes (CAZymes)^28^ were determined against the dbCAN2 database^29^ by using the HMMER model with a setting of e-value 0.00001. The virulence factors (VFs) were identified against a database for virulence factors (DFVF) rice blast database^30^ using DIAMOND (v0.9.26)^31^ protein aligner with parameters of max-target sequence alignment 1, ≥ 100 amino acid length, 90% identity, 60% query coverage, 60% subject coverage and e-value 0.00001. For prediction of potential effectors in the studied genomes, initially the signal peptide features were determined in the protein sequences and sequences were then submitted to EffectorP3.0 for prediction of effectors with default setting^32^. Further, OrthoVenn2 comparison was performed to decipher the common and unique effector in this genome as compared to other genomes^33^. In order to determine metabolic pathways, the predicted protein sequences were submitted to the KAAS webserver^34^ using search program GHOSTX against the reference pathway model *Pyricularia grisea* 70-15^35^. The presence of different promoters were identified using promoter2.0^36^ with default settings, which detected 7021 promoters. For the comparative study, the used genome were GCA_000002495 (*M. oryzae* 70-15), GCA_002368485.1 (*M. oryzae* GUY11) and GCA_002924685.1 (*M. oryzae* WBKY11).

### Analysis of variants and phyologenetic tree

For SNP determination, the high quality reads were mapped against *M. oryzae* 70-15 reference genome using burrows-wheeler alignment (BWA) maximal exact match (MEM) module^37^. The mapped reads were subjected for the removal of PCR duplicate reads using Mark duplicates of Picard tool (https://broadinstitute.github.io/picard/). Finally, variant calling was done using SAMtools^38^ package bcftools mpileup script with settings of phred quality score (Q) ≥ 25, minimum variant depth (DP) ≥ 25 and mapping quality ≥ 30. The identified SNPs were annotated to genomic region and various effects type using snpEff 5.0^39^. For determination of genomes similarity, the comparative whole genome alignment based phylogenetic tree was generated using progressiveMauve aligner which consider gene rearrangement, gain and loss mechanism^40^. For alignment we used RMg_Dl and other 13 public genomes accession were GCA_002924685.1 (WBKY11), GCA_002021675.1 (Mo-nwi-55), GCA_000969745.1 (MG01), GCA_000832285.1 (B157), GCA_002218355.1 (H08-1c), GCA_001936435.1 (MG10), GCA_003991345.1 (10,100), GCA_000734215.1 (4603.4), GCA_000292605.1 (P131), GCA_002368485.1 (GUY11), GCA_000002495.2 (70-15), GCA_000475075.1 (HN19311), GCA_000805855.1 (98-06). The revised assembled genome was deposited in the NCBI database with GenBank assembly accession number GCA_001853415.3.

## Results

### Genome assembly, genes identification, and assessment

**Figure 2:**
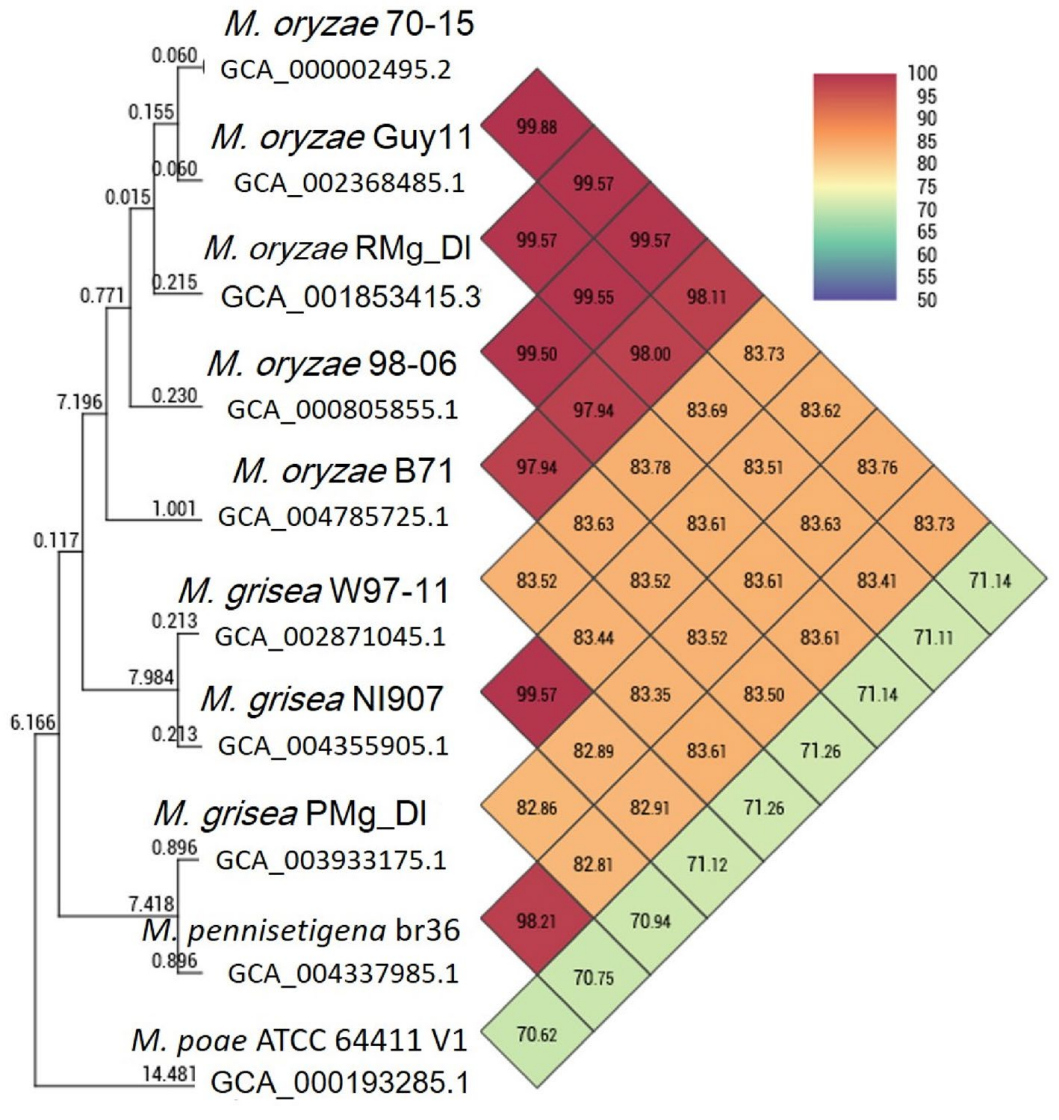
Average nucleotide identity (ANI) among various fungal *Magnaporthe/Pyricularia* genus

Using two of the prominent high-throughput whole-genome sequencing coupled with the hybrid assembly of short and long reads resulted in a total of 375 scaffolds with a genome size of 42.42 Mb (*M. oryzae* RMg_Dl). This genome-wide gene identification showed a total of 10,555 genes with 10,539 protein sequence features (Table 1). Additionally, comparative genome analysis revealed 99% average nucleotide identity (ANI) with other *M. oryzae* genomes and 83% against *M. grisea,* and 73% against *M. poe* genomes (Fig. 2). The quantitative genome assembly finish assessment showed that complete and single-copy BUSCO was 741 (97.76%) in *M. oryzae* (RMg_Dl) strain out of total 758 BUSCO genes. Although, we found thirteen missing BUSCO in *M. oryzae* RMg_Dl (Supplementary Table S1).

**Table 1:** Comparative statistics of the assembled whole genome of *M. oryzae* RMg_Dl.

Features	*M. oryzae* RMg_Dl
Illumina HiSeq-2500 reads (base)	65,731,812 pair-end (16.3 Gb)
PacBio-RSII reads (base)	192,820 single end (1.5 Gb)
Genome SIZE	42,437,481
Number of scaffolds	375
Scaffold N50	524,210
No of genes	10,553
Proteins	10,539
rRNA	73
tRNA	259
SSR	18,830
Virulence factors	51
CAZymes	542
Effectors	603

### Determination of transposable elements and SSRs

Determination of SSRs in RMg_Dl genomes showed that 11 different transposon families with a total of 10,250 copy numbers in the genome. The repeat types namely Type:EVERYTHING_TE occurred in the majority, whereas repeats such as DNA/TcMar-Fot1, LTR/Gypsy, LINE/Tad1, and Type:LINE is also more in copy number (Table 2). Further, SSR analysis showed that about 18,830 were present in RMg_Dl genome. Furthermore, in the case of di-nucleotides, (TA)_n_ was the most frequent, followed by (AG)_n_ and (AT)_n_. Similarly, among tri-nucleotide repeats, the (CAG)_n_ repeats were most frequent, followed by (GCT)_n_ repeats. In the case of frequency for tetra-nucleotide repeats, (TACC)_n_ was most frequent, followed by (TAGG)_n_ (Supplementary Table S2).

**Table 2:** The detected transposable elements in *M. oryzae* RMg_Dl genome.

Family	*M. oryzae* RMg_Dl
DNA	41
DNA/TcMar-Fot1	829
Type:DNA	870
LINE/Tad1	257
Type: LINE	257
LTR/Copia	344
LTR/Gypsy	1072
Type:LTR	1416
Type:EVERYTHING_TE	2543
Type:Simple_repeat	34
Type:Unknown	2587
Total	10,250

### Analysis of protein family and conserved domain in sequenced genomes

**Figure 3:**
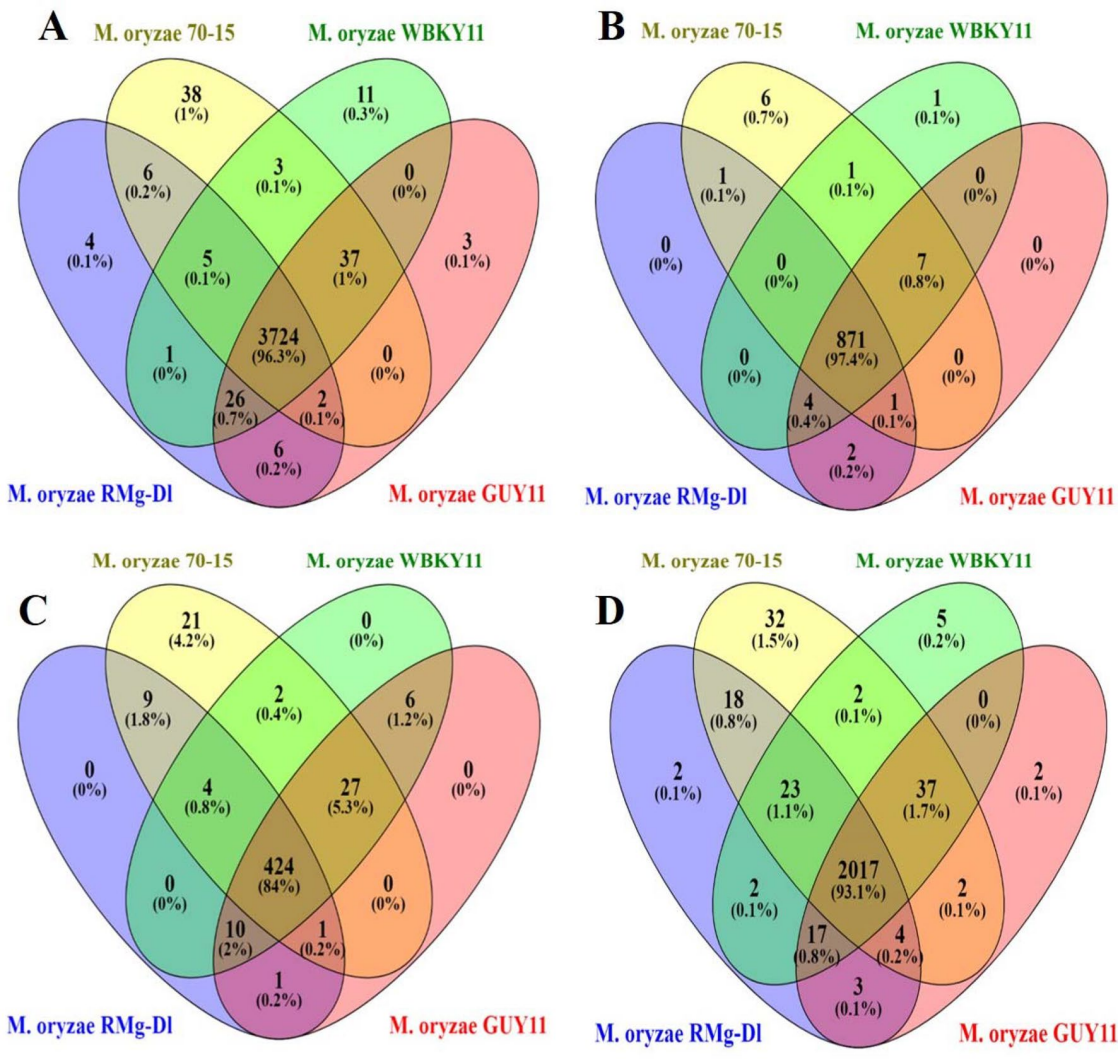
The predicted proteins InterProScan analysis of *M. oryzae* RMg_Dl, *M. oryzae* 70-15, *M. oryzae* WBKY11 and *M. oryzae* GUY11 genomes. The figure **A**, **B**, **C** and **D** shows the analyses against PFAM, superfamily, PIRSF and CDD databases, respectively.

The InterProScan analysis was performed for identifying protein family (PFAM) and superfamily, which showed the total occurrence of 3774 protein families, 879 superfamilies in the RMg_Dl genome. The comparison among RMg_Dl with genomes for shared and unique PFAM, protein information resource superfamily (PIRSF), superfamily, and conserved domains in sequenced genome showed that 3724 (96.3%) PFAMs, 871 (97.4%) superfamily, 424 (84%) PIRSF and 2017 (93.1%) CDD were shared in studied genomes (Fig. 3, Supplementary Tables S3, S4 and S5). The PFAM detailed exploration showed that family/domain such as WD domain, major facilitator superfamily, cytochrome P450, fungal specific transcription factor domain, protein kinase domain, and ABC transporter was highly prevalent (Supplementary Table S3). Similarly, superfamily identification showed that P-loop containing nucleoside triphosphate hydrolase, NAD(P)-binding domain, MFS transporter, Alpha/Beta hydrolase fold, FAD/NAD(P)-binding domain, and glycoside hydrolase superfamily were prevalent in the genome (Supplementary Table S4). Further, proteins were subjected for the transmembrane, signal peptide and cytoplasmic orientation classification using Phobius tool showed that transmembrane, non-cytoplasmic domain, and cytoplasmic domain associated proteins were highly present in the genome (Supplementary Table S6).

### Assembled genome functional annotation using gene ontology

**Figure 4:**
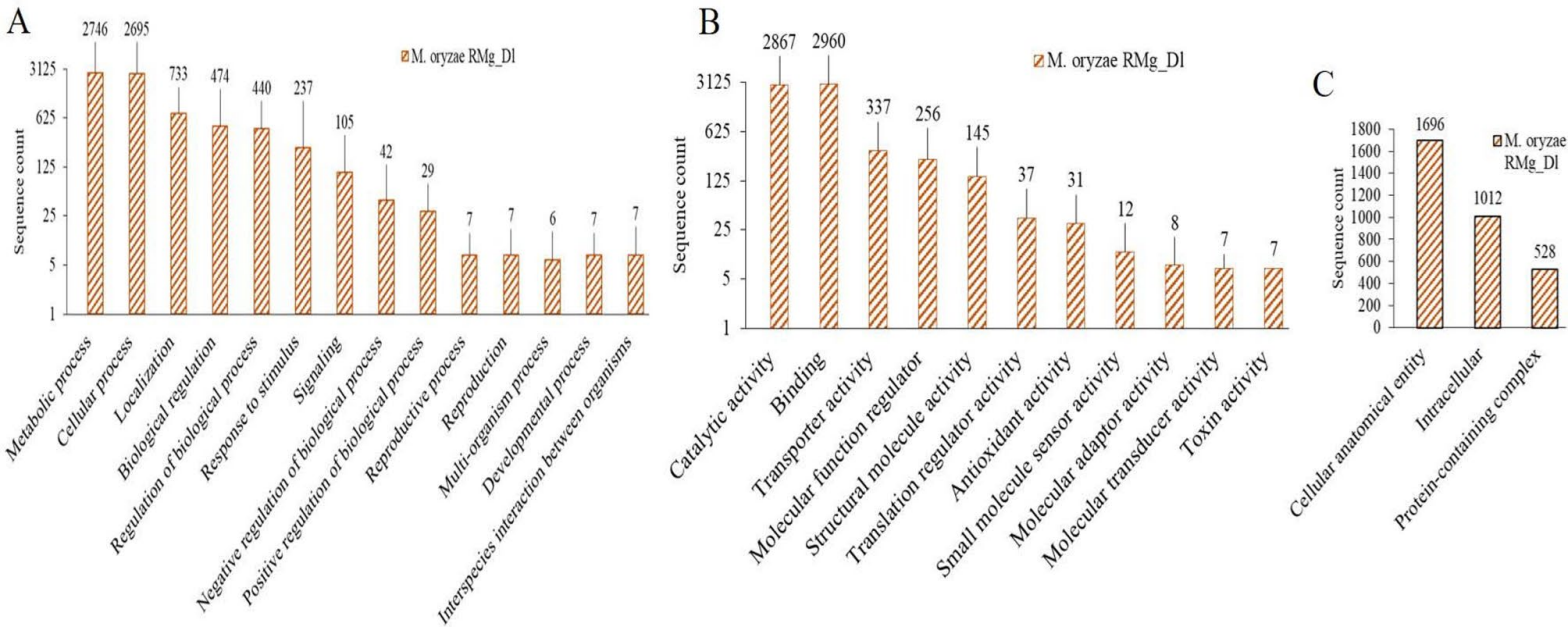
Functional annotation of predicted genes/proteins of *M. oryzae* RMg_Dl in GO term: biological process (**A**) and GO term: Molecular Function (**B**), and cellular component (**C**).

The functional annotations of genes predicted were performed based on gene ontology using InterProScan and Blast2GO mapping and annotation. It provides functional signature vocabulary and hierarchical network relationships for the gene products in three classes: biological process, molecular function, and cellular component. Gene ontology (GO) mapping and annotation of sequences resulted in enrichment at level 2 category of the biological process revealed that mapped sequences ranged between 2746 and 7, molecular function ranged from 2787 to 7. The sequences assigned for cellular components ranged from 1696 to 528 (Fig. 4). Among the biological process, the majority of genes were linked with metabolic processes, cellular processes, localization, biological regulation, signaling, negative and positive regulation of biological processes (Fig. 4A). The molecular function associated GO terms were highly prevalent for catalytic function, binding, transporter activity, molecular function regulator, and structural molecule activity (Fig. 4B). Similarly, a cellular component associated terms were cellular anatomical activity, intracellular and protein-containing complex were prevalent (Fig. 4C).

### Orthologous genes analysis

The orthologous features analysis for predicted proteins showed that 9216 orthologous genes were shared among RMg_Dl with 70-15, WBKY11 and GUY11 genomes, whereas 21 genes were found uniquely in RMg_Dl genome (Supplementary Fig. S1).

### Identification of pathogenicity genes, virulence factors (VFs) and effectors

**Figure 5:**
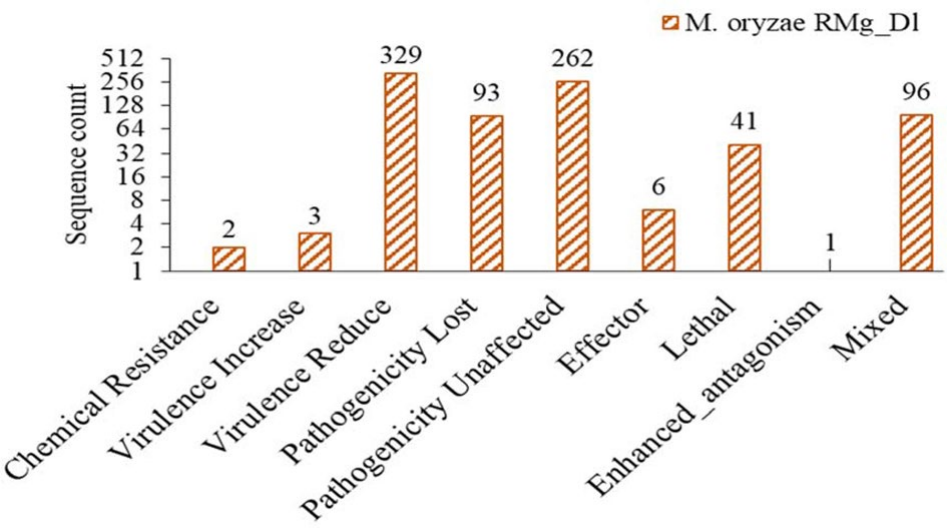
The assigned protein sequences to different PHI categories.

Further, the predicted proteins were analyzed against the pathogen-host interaction (PHI) database that revealed a total of 833 PHI genes were found in the RMg_Dl genome. Among that, the majority of PHI genes were associated with reduced virulence and pathogenicity loss (Fig. 5). Also, there were a total of 51 different VFs identified, and its annotation showed that four copies of these VFs identified as gypsy retrotransposon, and function like pathogenicity, toxin activity, etc. (Supplementary Table S7). Further, these VFs consist of protein families namely Sel1 repeat, Heat-labile enterotoxin alpha chain, and WD domain, G-beta repeat, and superfamily namely ADP-ribosylation, S-adenosylmethionine synthetase, etc. (Supplementary Table S8).

The effectors were predicted through phobius mapping which showed 1986 signal peptide sequences, and then EffectorP3.0 was utilized for possible potential effector determination. This showed that a total of 603 effector genes, in which 330 were cytoplasmic and 273 were apoplastic effectors (Supplementary Table S9). Thus, on an average, 5.72% of the total proteome content involved in effectors related functions. Upon the comparative study of these effectors with other genomes using orthologous approach, the total of 443 common effectors protein were documented, whereas 15 effectors were only shared among RMg_Dl and 70-15 genomes (Supplementary Fig. S2). Further, 10 effectors genes were identified as AVR proteins, which classified to AVR-Pii in majority (4 copy), followed by Avr-Pik (2 copy) and Avr-Pita, AVR-Pita2, Avr-Pi54, AVR-Pita1 with single copy. These effectors CAZymes annotation showed that a total of 31 families were found with its total 64 copies in this genome. Among that family, AA9 was found with high occurrence, followed by CE5, CE1, AA16, and GH11 etc., in identified effectors (Supplementary Table S10). Additionally, PFAM annotation showed that a total of 142 different PFAMs were identified, which accounted for 237 copies. Among that glycosyl hydrolase family-61 was the most abundant followed by fungal cellulose binding domain, and chitin recognition protein etc. (Supplementary Table S11).

### Identification of CAZymes

**Figure 6:**
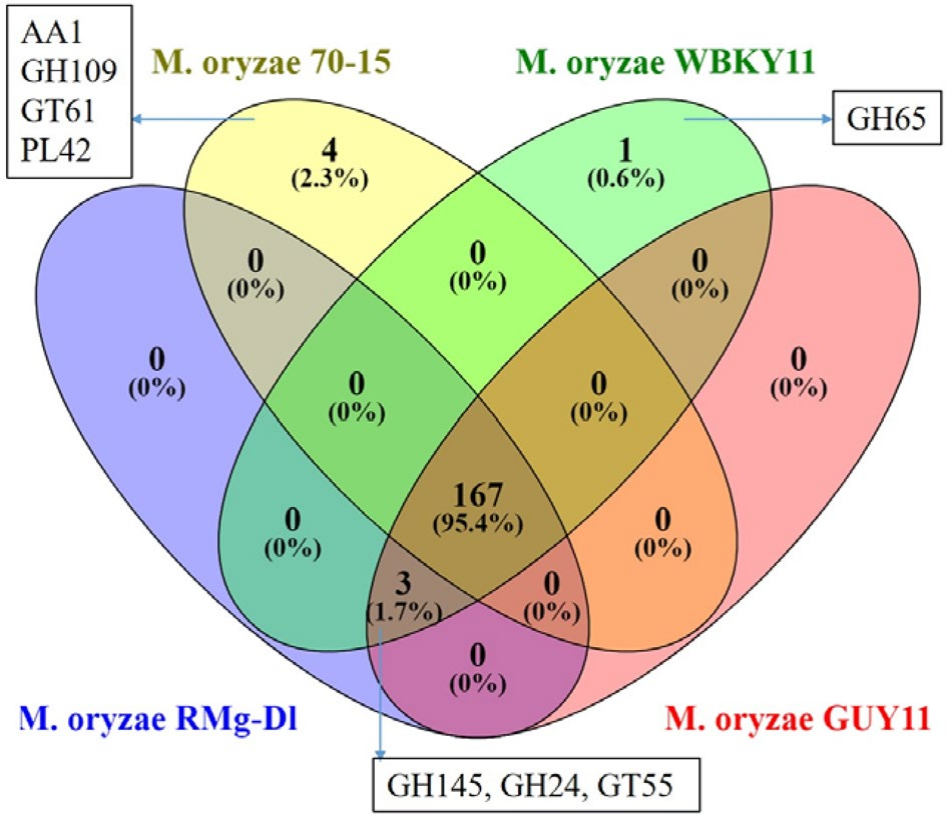
Identification and comparison of unique and shared CAZymes in *M. oryzae* RMg_Dl, *M. oryzae* 70-15, *M. oryzae* WBKY11 and *M. oryzae* GUY11 genomes.

The CAZymes identification showed the presence of different 542 CAZyme families. These CAZymes extended analyses showed that the most abundant family was glycoside hydrolase (GH) (257 GH family). Next to GH, the auxiliary activities (AA) was the second-highest family (118 AA). The third highest abundant family was glycosyltransferase (GT) (94 GTs). Interestingly, the pectin lyase (PL) family showed the least abundance in the CAZyme family composition (Table 3). Further, the downstream analysis revealed that the AA7 and AA9 family was the most abundant, followed by GH3 and CE5 (Supplementary Table S12). Additionally, the other highly abundant families such as CE4, GH47, GH2, AA11, CE3, GH131, GH31 were found to be equally present in studied genomes. Overall, a comparison of all CAZymes studied in genomes revealed that 167 (95.4%) families were shared between RMg_Dl, 70-15, WBKY11 and GUY11 genomes. Although, none of the CAZymes were found to be uniquely present in RMg_Dl whereas AA1, GH109, GT61, and PL42 families were uniquely detected in the 70-15 genome (Fig. 6).

**Table 3:** The comparative CAZymes family profile in *M. oryzae* RMg_Dl, *P. oryzae* 70-15, *M. oryzae* WBKY11, and *M. oryzae* GUY11 genomes.

Family	*M. oryzae* RMg_Dl	*P. oryzae* 70-15	*M. oryzae* WBKY11	*M. oryzae* GUY11
AA	118	130	121	115
CBM	14	14	14	14
CE	53	53	54	53
GH	257	256	263	260
GT	94	100	98	94
PL	6	7	7	7
Total	542	560	557	543

### Identification of metabolic pathways

**Figure 7:**
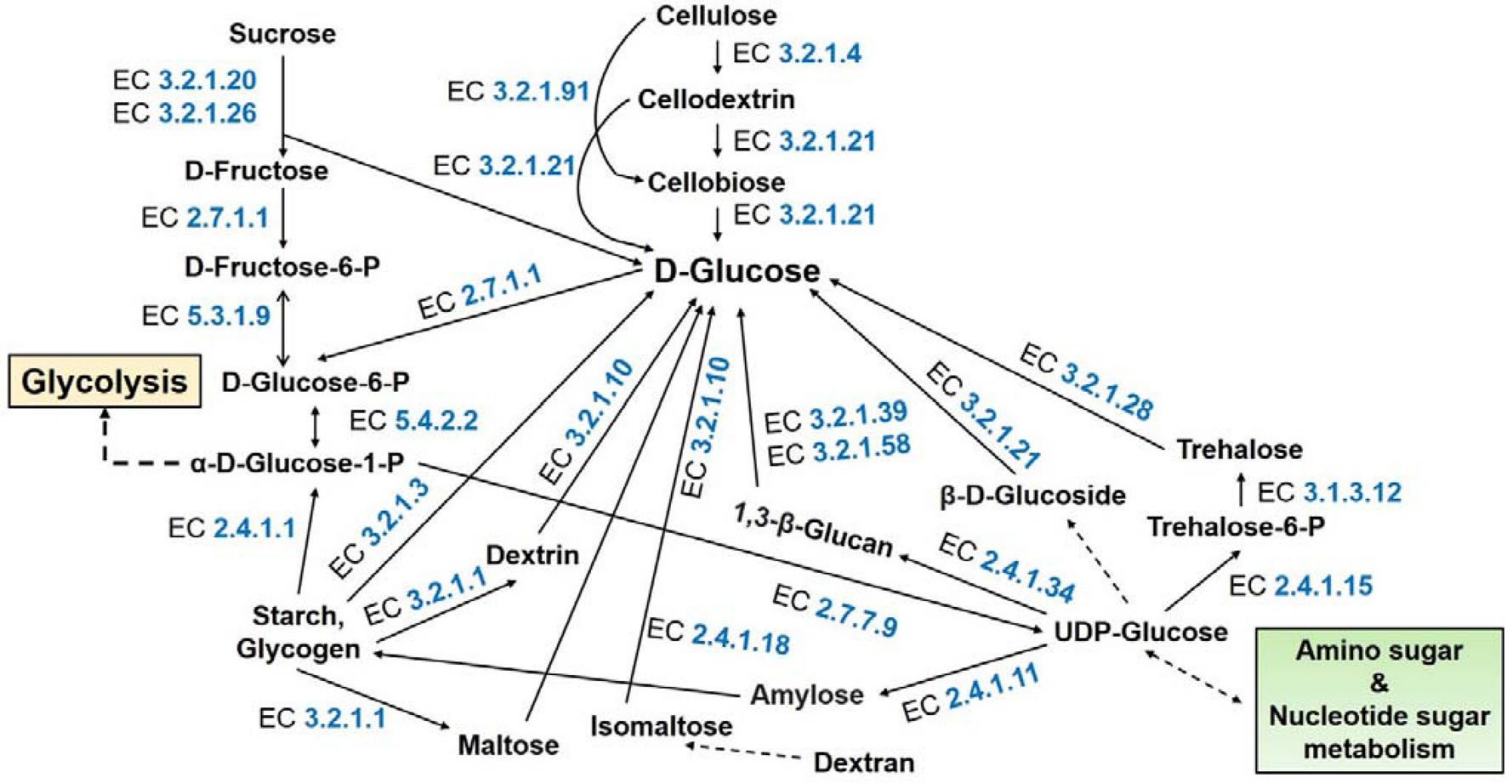
Starch and sucrose metabolism pathways depicting various enzymes involved including plant cell wall degrading enzymes. In here, the Sysname of enzymes related to the biosynthetic machinery are depicted in blue-colored EC numbers: The key to all of the EC numbers are as follows: EC 3.2.1.4, 4-β-D-glucan 4-glucanohydrolase; EC 3.2.1.21, β-D-glucoside glucohydrolase; EC 3.2.1.91, 4-β-D-glucan cellobiohydrolase (non-reducing end); EC 3.2.1.20, α-D-glucoside glucohydrolase; EC 3.2.1.26, β-D-fructofuranoside fructohydrolase; EC 2.7.1.1, ATP:D-hexose 6-phosphotransferase; EC 5.3.1.9, D-glucose-6-phosphate aldose-ketose-isomerase; EC 2.4.1.15, UDP-α-D-glucose:D-glucose-6-phosphate 1-α-D-glucosyltransferase; EC 3.1.3.12, α, α-trehalose-6-phosphate phosphohydrolase; EC 3.2.1.28, α, α-trehalose glucohydrolase; EC 2.4.1.11, UDP-α-D-glucose: glycogen 4-α-D-glucosyltransferase; EC 2.4.1.18, (1->4)-α-D-glucan:(1->4)-α-D-glucan 6-α-D-[(1->4)-α-D-glucano]-transferase; EC 2.4.1.1, (1->4)-α-D-glucan:phosphate α-D-glucosyltransferase; EC 3.2.1.1, 4-α-D-glucan glucanohydrolase; EC 3.2.1.3, 4-α-D-glucan glucohydrolase; EC 3.2.1.10, Oligosaccharide 6-α-glucohydrolase; EC 5.4.2.2, α-D-glucose 1,6-phosphomutase; EC 2.4.1.34, UDP-glucose:(1->3)-β-D-glucan 3-β-D-glucosyltransferase; EC 3.2.1.39, 3-β-D-glucan glucanohydrolase; EC 3.2.1.58, 3-β-D-glucan glucohydrolase; EC 3.2.1.21, β-D-glucoside glucohydrolase and EC 2.7.7.9, UTP: α-D-glucose-1-phosphate uridylyltransferase.

**Figure 8:**
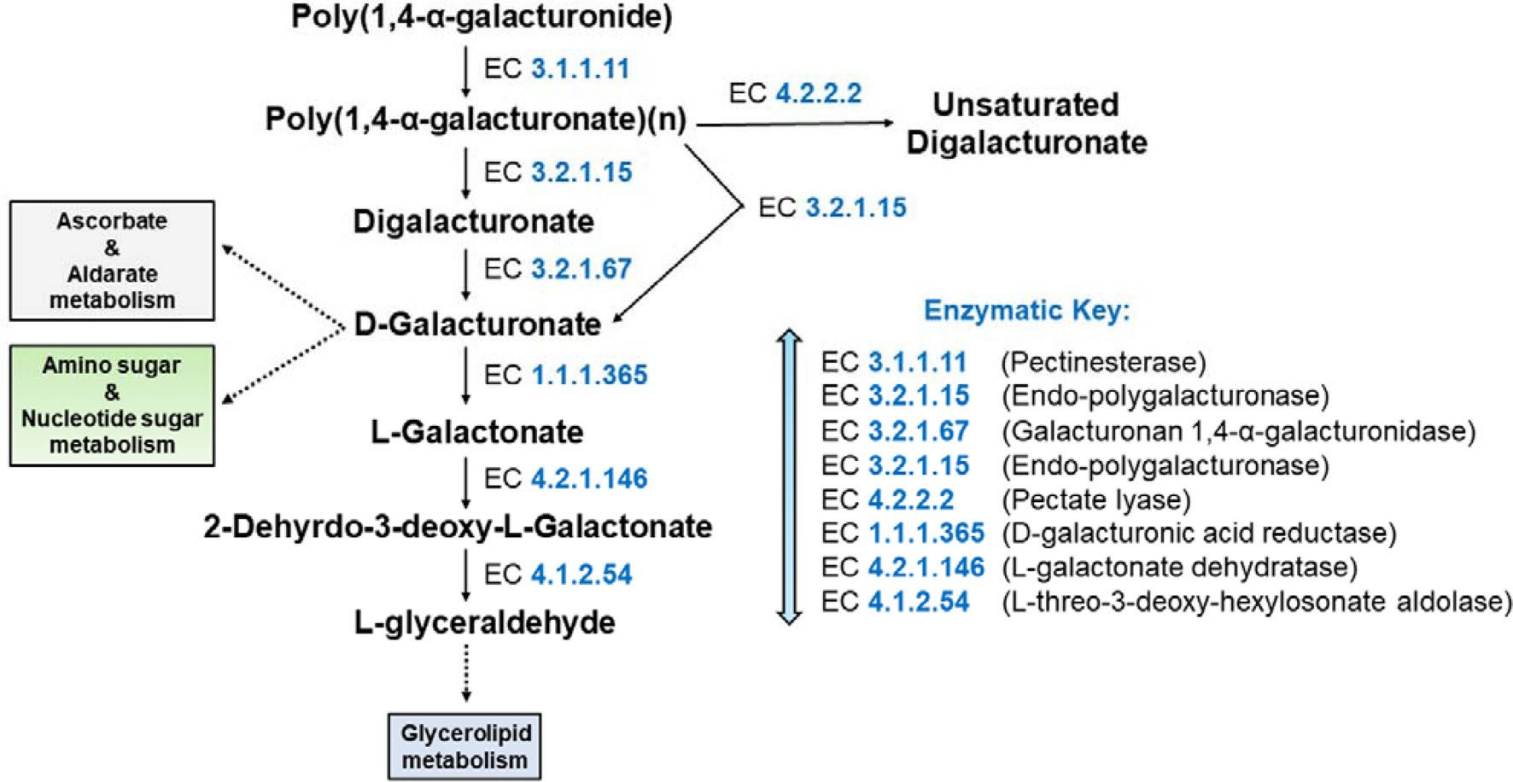
Pentose and glucuronate interconversion depicts the route enzymes of plant cell wall degrading enzymes.

The KEGG metabolic pathway analysis of the sequenced genome showed that the majority of genes were linked with metabolism and cellular process biochemical metabolic pathway categories. The detected pathways namely metabolism (KO:09100), in particular, carbohydrate metabolism (KO:09101) associated genes were 282 in the genome, and these genes were found to be distributed in fifteen different biochemical pathways (Table 4). Additionally, various genes are linked with pathways such as glycolysis/gluconeogenesis, starch, and sucrose metabolism, butanoate metabolism, propanoate metabolism, and inositol phosphate metabolism, fructose, and mannose and metabolism, pentose phosphate pathway were detected in the genome (Fig. 7, Supplementary Fig. S2). Among them, plant polysaccharide namely cellulose degradation key pathway, starch, and sucrose metabolism route-main enzyme endoglucanase (EC 3.2.1.4), cellulose 1,4-beta-cellobiosidase (EC 3.2.1.91), and beta-glucosidase (EC 3.2.2.21) were found in the genome. Similarly, pentose and glucuronate interconversions pathways involve the pectin degradation enzyme endo-polygalacturonase (EC 3.2.1.15) and pectinesterase (EC 3.1.1.11) and pectate lyases (polygalacturonate lyase) (EC 4.2.2.2) were also found in this genome (Fig. 8, Supplementary Fig. S3). Analysis of the biochemical pathways associated with energy metabolism (KO:09102) resulted in distinguishing total of 134 genes distributed in six (6) different pathways. Among the pathways, the majority of the genes were associated with oxidative phosphorylation followed by sulfur and nitrogen metabolism (Supplementary Table S13).

**Table 4:** Carbohydrate metabolism KEGG (Kyoto Encyclopedia of Genes and Genomes) pathway for *M. oryzae* RMg_Dl.

Carbohydrate metabolism pathways	*M. oryzae* RMg_Dl (Gene count)
KO:00010 Glycolysis / Gluconeogenesis	26
KO:00020 Citrate cycle	21
KO:00030 Pentose phosphate pathway	17
KO:00040 Pentose and glucuronate inter conversions	19
KO:00051 Fructose and mannose metabolism	20
KO:00052 Galactose metabolism	15
KO:00053 Ascorbate and aldarate metabolism	5
KO:00500 Starch and sucrose metabolism	26
KO:00520 Amino sugar and nucleotide sugar metabolism	27
KO:00620 Pyruvate metabolism	29
KO:00630 Glyoxylate and dicarboxylate metabolism	23
KO:00650 Butanoate metabolism	12
KO:00640 Propanoate metabolism	18
KO:00660 C5-Branched dibasic acid metabolism	3
KO:00562 Inositol phosphate metabolism	21
Total	282

The identification of environmental information processing (KO:09130) pathways revealed that 404 genes were in-particularly associated with signal transduction (KO:09132) pathways. The identified genes were involved in a total of thirty-two biological (biochemical) pathways (Table 5). These signaling pathways extended exploration showed that MAPK signaling pathway (Supplementary Fig. S4) associated genes were highest, followed by mTOR signaling pathway (KO:04150), PI3K-Akt signaling pathway (KO:04151), and AMPK signaling pathway (KO:04152). The other detected pathways were the two-component system, RAS signaling pathway, and calcium (Ca^2+^) signaling pathway, etc.

**Table 5:** Signal transduction KEGG (Kyoto Encyclopedia of Genes and Genomes) pathway for M*. oryzae* RMg_Dl.

Signal transduction pathways	*M. oryzae* RMg_Dl (gene count)
KO:02020 Two-component system	19
KO:04014 Ras signaling pathway	15
KO:04015 Rap1 signaling pathway	6
KO:04010 MAPK signaling pathway	15
KO:04013 MAPK signaling pathway-fly	13
KO:04016 MAPK signaling pathway-plant	3
KO:04011 MAPK signaling pathway-yeast	56
KO:04012 ErbB signaling pathway	5
KO:04310 Wnt signaling pathway	13
KO:04330 Notch signaling pathway	4
KO:04340 Hedgehog signaling pathway	5
KO:04341 Hedgehog signaling pathway-fly	7
KO:04350 TGF-beta signaling pathway	6
KO:04390 Hippo signaling pathway	9
KO:04391 Hippo signaling pathway-fly	7
KO:04392 Hippo signaling pathway-multiple species	6
KO:04370 VEGF signaling pathway	7
KO:04371 Apelin signaling pathway	12
KO:04630 Jak-STAT signaling pathway	4
KO:04064 NF-kappa B signaling pathway	4
KO:04668 TNF signaling pathway	2
KO:04066 HIF-1 signaling pathway	15
KO:04068 FoxO signaling pathway	13
KO:04020 Calcium signaling pathway	9
KO:04070 Phosphatidylinositol signaling system	17
KO:04072 Phospholipase D signaling pathway	12
KO:04071 Sphingolipid signaling pathway	19
KO:04024 cAMP signaling pathway	9
KO:04022 cGMP-PKG signaling pathway	8
KO:04151 PI3K-Akt signaling pathway	24
KO:04152 AMPK signaling pathway	23
KO:04150 mTOR signaling pathway	37
Total	404

### Identification of variants

**Figure 9:**
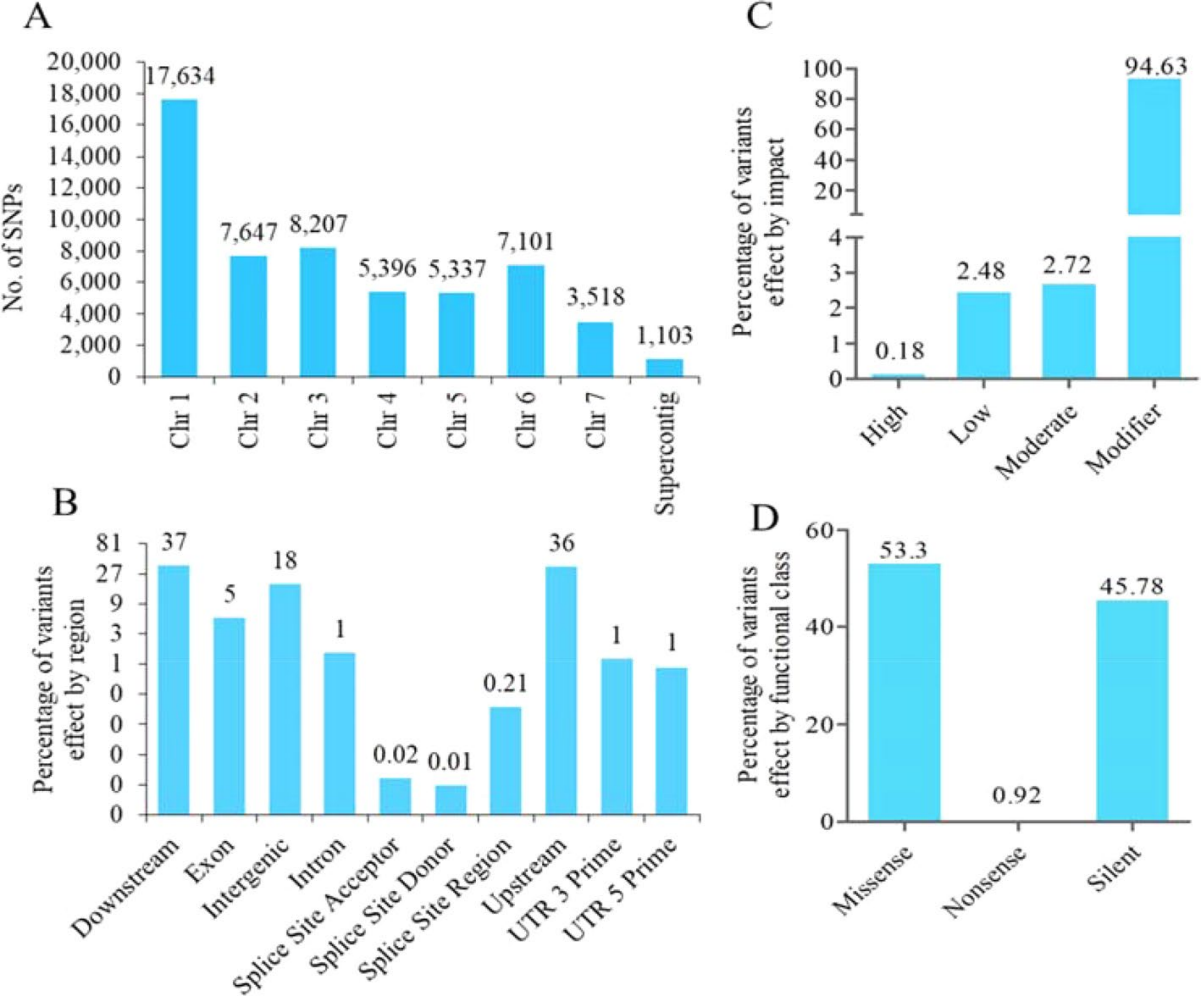
Identified variants and effect annotation of *M. oryzae* RMg_Dl genome. **A** = variants distribution by chromosome, **B** = effect by different region, **C** = effect by impact and **D** = effect by functional class.

The SNPs and InDels calling showed that a total of 55,943 variants with their distribution as 49,065 SNPs, 3267 insertions and 3611 deletions in this genome. The SNPs chromosome wise classification showed that the highest SNPs were found on chromosome 1 and followed to chromosome 3 (Fig. 9A). The genome wide distribution of SNP density determination showed the occurrence of ~14 SNPs per 10 Kb in *M. oryzae* RMg_Dl genome. The identified SNPs transition and transversions were 39,326 and 9738, with their transition–transversion (Ts/TV) ratio was 4.04. The annotation of variants classified effects to region-wise, impact and functional class (Fig. 9). The majority of identified variants effect by region-wise were downstream (37%), followed to upstream (36%) and intergenic (18%) (Fig. 9B). The effect by impact showed that majority of variants depicted modifier (94.63%) followed to moderate (2.72%) and least for high (0.18%) impact (Fig. 9C). Moreover, effect by functional class, the missense type variants were 53.3% and silent were 45.78% (Fig. 9D). The conservative in-frame insertions and deletion were 0.05% and 0.04%, respectively. Similarly disruptive in-frame insertion and deletion were 0.03% and 0.03% respectively (Supplementary Table S14). Since the *M. oryzae* is a kind of rice blast disease causing pathogenic fungi, therefore we extensively performed the occurrence of variants in virulence factors and predicted potential effectors. Among the detected VFs, the total of 1340 variants were identified, in which 115 SNPs were identified as missense type in 7 different VFs. Also, majority of identified missense SNPs of VFs were found on chromosome 1, followed by chromosome 6, and VF namely *MGG_09263* was found with highest SNPs (82) (Supplementary Table S15). Similarly, for effectors, the total of 6743 variants were identified, among that 5768 were SNPs and 975 were indels. Effectors missense type SNPs determination showed that a total of 71 missense type SNPs, with highest occurrence on chromosome 1 followed by chromosome 4 and 2. The effector *MGG_17020* identified with highest missense SNPs, which was located on chromosome 4 (Supplementary Table S16). The occurrence of variants in AVR effectors were 75 in which 63 were SNPs and 12 were InDels. Among this, most of the variants were located in upstream region. The single missense SNP was was found in Avr-Pii, and located on Chromosome2: 842166G > A (c.188C > T, p. Ala63Val). The effectors conservative and disruptive inframe InDels annotation showed that total of 29 InDels, in which conservative inframe deletion and insertions were 10 and 6, whereas disruptive inframe deletion and insertion were 6, and 7 respectively (Supplementary Table S17).

## Discussion

The advent of high throughput sequencing technologies with short and long-read sequencing chemistry coupled with hybrid assembly have extensively facilitated in depth understanding of biochemical pathways, virulence factors, and conserved domains in the fungal genomes. In this study, we performed the WGS of rice blast disease-causing fungal species namely *Magnaporthe oryzae* RMg_Dl. The  blast fungus is one of the wide spread pathogen reported to cause epidemics in different geographical locations and all major rice varieties^6,41,42^. Therefore, the comparative whole genome alignment based phylogenetic tree was generated to decipher the evolutionary relationship of RMg_Dl with other 13 *Magnaporthe* genomes publically released from India, Japan, China, Thailand (Asia), USA and Guyana (Fig. S6). This analysis depicted the close relationship of Indian genomes with each other (Cluster 1), whereas moderately related to isolates representing Japan, Thailand, and shared distant relationship with Guyana (GUY11), USA (70-15) and China (HN19311, 98-06) genomes (Cluster 3). This indicated the *M. oryzae* pathotypes representing same or proximal geographic locations (Asian continent) showed high genetic similarity (India, China, Thailand and Japan), except WBKY11 (USA) genome. In our previous study, we reported that diverse pathotypes of *Magnaporthe* were genetically homogenous indicating the trans-boundary movement across the continents^43^. Such a pathogenic variation could be possibly associated with dynamic mechanism such as genetic mutations, recombination, geographical location and host resistance.

 In the sequenced genome, we identified various genes involved in host–pathogen interaction such as virulence and pathogenicity mediators. Such occurrence of pathogenicity-related genes plays an essential role in initiating infection in the host^42–44^. These genes mutation is believed to confer resistance against fungicides^45^. Additionally, these genes have been documented for conferring fungicides resistance at the field level^46^. In this study, we identified 51 different VFs containing 59 PFAM and 35 superfamilies, with four VFs identified as gypsy retrotransposon. The VFs were reported for functions like pathogenicity, toxin activity, etc. The NR database blast classified VFs namely natural trehalases acts as trehalose breakdown (component of plant cell wall) and regulated by signaling pathway and activation by phosphorylation and stimulated by Ca^2+^ and Mn^2+^^47^. Similarly another VFs encode ‘ras-like protein ced-10’ involved in nutrient availability sensing and linked with cell growth and morphogenesis^48^. The gypsy retrotransposon reported for DNA segment transposition, re-arrangement, genome evolution and widely distributed in ascomycota fungi and more details described here^49^.

Among the total effectors, majority of effectors were cytoplasmic which acts initially upon infection enriches in biotrophic interfacial complex (BIC) prior getting transfered into plant cells, and continues its secretion after invasive hyphae development and invade adjacent plant cells^50^. Whereas, apoplastic effectors upon secretion, disseminated in the extracellular space of the fungal cell wall and extra-invasive-hyphal membrane (EIHM). These secreted effector are a small secreted proteins that alter host cell organization and function like manipulate plant immunity and physiology to promote infection through suppressing or activating effector-triggered immunity (ETI)^51^. The AVR-Pii and AVR-Pik were reported for genomic instability^52^. AVR-Pii, Avr-Pita effector reported for damaging the host innate immunity^53^. Functional annotation of effectors using PFAM revealed that total of 237 families, but there are decreased annotation compared to total effectors, because possibly most of the effectors were classified as putative uncharacterized/hypothetical proteins. Therefore, utilizing the resources such as protein domains, families and superfamily driven classification could provide more insights of possible functional features of proteins and thus predicted effectors also^26^. Using this approach, the documented effectors classification showed that higher occurrence of glycosyl hydrolase family 61, fungal cellulose binding domain and cutinase were observed. In particular, cutinase family associated with diverse functions such as cell surface recognition, germinal differentiation, appressorial growth, host infiltration and then virulence maturation. Moreover, role of this protein documented for cyclic AMP/protein kinase A and diacylglycerol protein kinase C signalling upon activation which eventually triggers appressorium development and infection progress by this fungus^54^.

Moreover, we identified a total of 49,065 SNPs, 3267 insertions and 3611 deletions in this genome with ~14 SNPs per 10 Kb. The majority of variants were found on chromosome 1 and similar result was reported for other *M. oryzae* strains^55^. Further, virulence associated SNPs were located on chromosome 1, 2, 3, 4 and 7^56^. In this study, we found various SNPs on these chromosomes. The variants annotation effects classified into region wise effect showed that majority of variants were located in the gene regulatory elements namely downstream and upstream regions. The occurrence of variants in these regions potentially invovlve in gene expression regulation resulted in upregulation or downregulation. Interestinglly, we observed various missense SNPs which causes the amino acid change in protein structure resulting to altered protein property and functionality. This could be also associated with prompting infection, severity and susceptibility with increased incidence of fungal disease^57^. We documented various SNPs among genomic regions of upstream, downstream, and untranslated region (UTR) region, which influence the gene expression and regulation at the post-transcriptional level and protein synthesis^58^. The 5′UTR mutation influence the binding of proteins and results to stimulation or suppression of translational regulation. Meanwhile 3′UTR mutation reported to influence the binding sites of miRNA and polyA which affect the translational deregulation^59^. In this study, 54 effectors genes were documented with missense and InDels genetic variations, which accounted nearly 0.09% (54/603) of total effectors genes. Though, there were a single missense SNPs was found on Avr-Pii effector. Also we found various SNPs in AVR-Pita1 and AVR-Pita2, and previously reported for high variability and its diversification^60^.

The study of genomes for families, conserved domains through InterProScan resulted in the identification of nearly 96% PFAM, 84% PIRSF, and 93% CDD were shared in the studied genomes. Further, in the present study, the majority of PFAMs namely WD domain, G-beta repeat, major facilitator superfamily, and cytochrome P450 family, P-loop containing nucleoside triphosphate hydrolase, NAD(P)-binding domain, and alpha/beta hydrolase fold superfamily were expanded the genome. These detected families and conserved domains were documented for various mechanisms viz. MAPK for growth^61^, virulence, and pathogenicity^62^, ABC transporter for virulence^63^ that overall mediate multiple significant roles in pathogen-host interaction^64^.

Repeats are also unique features of fungal genomes, and these identified SSRs could be utilized for fungal diversity study and disease management, species/strain differentiation, and detection^65^. The rich genomic bases of GC and AT are applied for the assessment of fungal defense against transposon expansion which works through repeat-induced point mutation (RIP) and genome evolution. Such processes play a differentiating choice and facilitation of host-microbe interaction^66^. In this study, we documented more than 18,000 SSRs which can be applied for the study of fungal population diversity and potential application towards making an efficient management strategy for disease prevention. Such methods are efficiently applied for controlling the citrus leaf and fruit disease caused by fungi^67^.

The study on fungal genomes about the content of CAZymes showed that this organism contains distinct types of CAZyme families, which facilitates fungus to efficiently degrades host complex polysaccharides. The CAZymes also play an essential role in pathogen-host interactions (PHI) as pathogenic fungus invades host on plant cell wall via the action of cell wall degrading enzymes^12^. We detected the high occurrence of GH families, and studies reported that the GH family plays a potential role in the breakdown of complex polysaccharides coupled with additional CAZymes like CBM and PL, which increase the breakdown efficacy and pave the path for other breakdown processes in the environment^69^. Moreover, the genomic study of various pathogenic fungal species showed vast heterogeneity in CAZymes content because of their host specificity and nutritional requirement, which also facilitated the digestion of complex plant polysaccharides for nutritive addition and later simplify the infection process^12,70^. The pathogenic fungus is reported to contain a distinct number of CAZymes, whereas less than saprophytic and necrophytic fungi^12^. The high content of various GH family also reported in fungus invading the monocot than dicot plant because monocot enriched with polysaccharides^12,70^.

The biochemical pathway determination detected the presence of various metabolic pathways associated with variable enzymes in the genome. Among that plant polysaccharide namely cellulose degradation key pathway, starch and sucrose metabolism route-main enzyme endoglucanase (EC 3.2.1.4), cellulose 1,4-beta-cellobiosidase (EC 3.2.1.91), and beta-glucosidase (EC 3.2.2.21) were found in the genome. The endoglucanase (EC 3.2.1.4) initially degrades cellulose to cellodextrin and then to cellobiose. Cellulose 1,4-beta-cellobiosidase (EC 3.2.1.91) degrades i) cellulose to cellobiose, ii) cellodextrin to cellobiose. Then, beta-glucosidase (EC 3.2.2.21) generates glucose via two ways- i) cellobiose to glucose, and ii) cellodextrin to glucose (Fig. 7). Further cell wall degrading enzyme endo-polygalacturonase (EC 3.2.1.15) of PL29 CAZymes, and pectate lyases (EC 4.2.2.2) of PL1 CAZymes and endo-1,4-beta-xylanase (EC:3.2.1.8) were detected which further mediate a typical role in infection and utilizes it for own growth and reproduction^71,72^. These enzymes are recognized for cell wall degradation enzyme (CWDE) which facilitates the infection and plant cell damage^70,71^. The enzymatically released glucose and other compounds were utilized by fungi for their growth, reproduction, and proliferation in the host^71^.

Functional pathway two-component system also plays an essential role in rice blast disease incidence along with the combined mechanism of MAP kinase and cAMP signaling mediated formation of infection component on rice host^62,73^. Reports also demonstrated that cAMP signaling was associated with fungal growth and infection development^73–75^. Such kinase signaling mechanisms include the phosphorylation mediated signaling process, chemotaxis, virulence process, and secondary metabolite production in fungi^76^. Additionally, the mechanism of histidine kinase-mediated environmental responses, pathogenicity, hyphal development, and then sporulation have also been documented^8,77,78^. The MAPK pathway descriptive study revealed that various enzymes of *M. oryzae* RMg_D1 were mapped to signaling mechanisms such MAPKKK, MAPKK, and MAPK (Supplementary Fig. S4). Additionally, the protein domains are linked to each other for creating multi-domain protein structures to gain a wide range of functional property^79,80^. This can be exemplified through flexible architectures of signaling pathways like mitogen-activated protein kinase (MAPK) cascades^81,82^. This pathway is associated with controlling for various biological processes such as metabolism, cellular morphology, cell cycle progress, and gene expression in the influence of any extracellular signals or stimuli^83^ and cellular signaling and pathogenesis-related structure development^64,84^. In particular, among pathogenic fungi, metabolic pathways, ATP-binding cassette (ABC) transporters are primarily involved in defense activity against secondary metabolites or toxins secreted by the host^85^.

 In this sequenced genome, we observed various transport families such as the ABC transporter, phosphate transporter family, major facilitator superfamily (MFS) involved in the transport of a broad range of minerals and nutrients. The role of ABC and MFS transporters documented for the protection against natural toxic substance exists in the atmosphere or synthetic toxic compounds such as fungicides and antimycotic agents^41,86^. Pitkin, et al.^87^ reported that ABC transporters also offer defense against antimicrobial agents. Additionally, crucial role in host pathogenicity while providing protection against host defense mechanisms or releasing host-specific toxins. These transporters play as a resistance barrier against various fungicides, though prolonged uses of fungicidal agents resulted in the occurrence of resistance in ABC and MFS transporter including chemically unrelated agents and causes the decreased accumulation of these agents^41,86^. We also detected superfamily namely cytochrome P450 (CYP) monooxygenase superfamily which is reported to be involved in a wide range of functions such as multidimensional metabolic activity and support to survival in a distinct ecological environment^88^ with a contribution in infection occurrence. The fungal CYP associated with distinct kind of secondary metabolites production for its own protection and compete against attacking organism such as bacteria, plants, animals and also against other fungi^89,90^. These compounds act as wide range of beneficial role like antibiotic, immune suppressor and mycotoxic actions^91,92^.

## Conclusion

In the present study, the whole genome sequencing of filamentous rice blast disease-causing fungus *M. oryzae* RMg_Dl was performed. The functional annotation revealed the presence of distinct enzymes linked with various metabolic pathways. The study also documented various carbohydrate metabolism-associated pathways that included starch and sucrose metabolism, pentose and glucuronate interconversion, and signaling associated namely MAPK, cAMP pathways. We also observed various CAZymes with high content of the GH family, pathogen-host interaction-related genes, effectors and virulence factors. This information serves as a genomic architecture for the optimization of genus *Magnaporthe* mediated blast disease management and assessment of population diversity. Moreover, detected genes or proteins can be utilized as potential targets for marker development to screen the blast pathogenic organisms.

## Supplementary Information

Supplementary Figures.

Supplementary Tables.

Supplementary Tables.
