# Supplementary material for: Hybrid de novo genome-reassembly reveals new insights on pathways and pathogenicity determinants in rice blast pathogen Magnaporthe oryzae RMg_Dl
Source: Sci Rep. 2021 Nov 25;11:22922. doi: 10.1038/s41598-021-01980-2 (PMC8616942; doi:10.1038/s41598-021-01980-2)
Supplement: Supplementary file 1 — Supplementary Figures. [file 41598_2021_1980_MOESM1_ESM.docx]

**Supplementary file**

**Scientific Reports**

**Hybrid *de novo* genome-reassembly reveals new insights on pathways and pathogenicity determinants in rice blast pathogen *Magnaporthe oryzae* RMg_Dl**

Bhaskar Reddy^1^, Aundy Kumar^1^*, Sahil Mehta^3^, Neelam Sheoran^1^, Viswanathan Chinnusamy^2^, Ganesan Prakash^1^

^1^Division of Plant Pathology, ICAR-Indian Agricultural Research Institute, New Delhi-110012, India

^2^Division of Plant Physiology, ICAR-Indian Agricultural Research Institute, New Delhi-110012, India

^3^Crop Improvement Group, International Centre for Genetic Engineering and Biotechnology, New Delhi-110067, India

***Corresponding author:** [kumar@iari.res.in](mailto:kumar@iari.res.in) (Aundy Kumar),

Orcid ID: [https://orcid.org/0000-0002-7401-9885](https://orcid.org/0000-0002-7401-9885?lang=en) (Aundy Kumar),

<https://orcid.org/0000-0002-8177-9305> (Bhaskar Reddy)

**
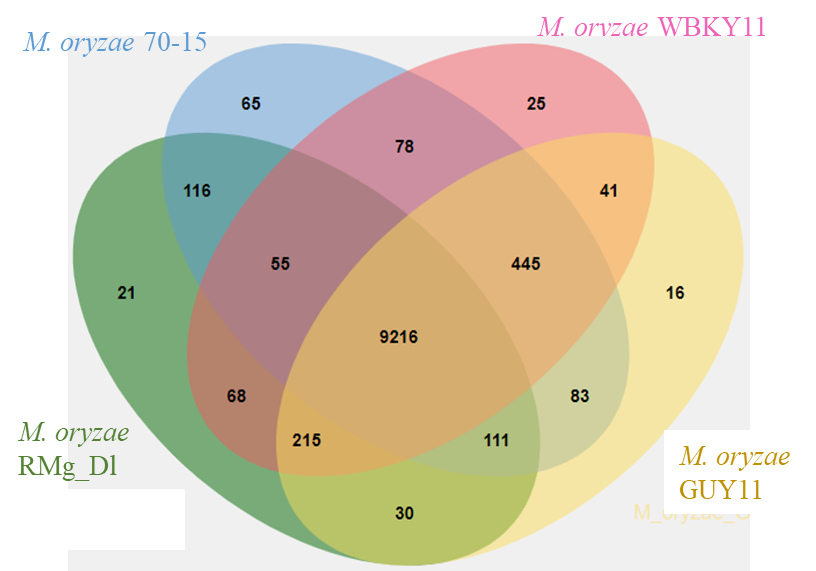
**

**Supplementary Fig. S1.** The Venn plot showing orthologous genes among the *M. oryzae* RMg_Dl, *M. oryzae* 70-15, *M. oryzae* WBKY11 and *M. oryzae* GUY11 reference genomes.


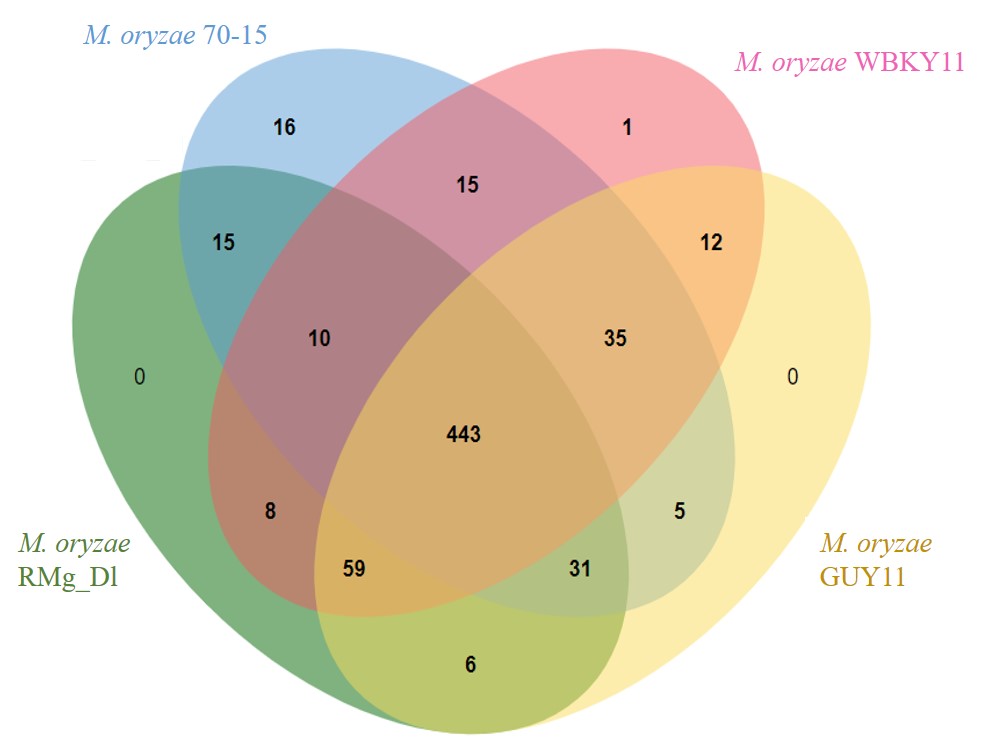


**Supplementary Fig. S2.** Venn plot showing the predicted effectors common and unique orthologous proteins among the *M. oryzae* RMg_Dl, *M. oryzae* 70-15, *M. oryzae* WBKY11 and *M. oryzae* GUY11 reference genomes.


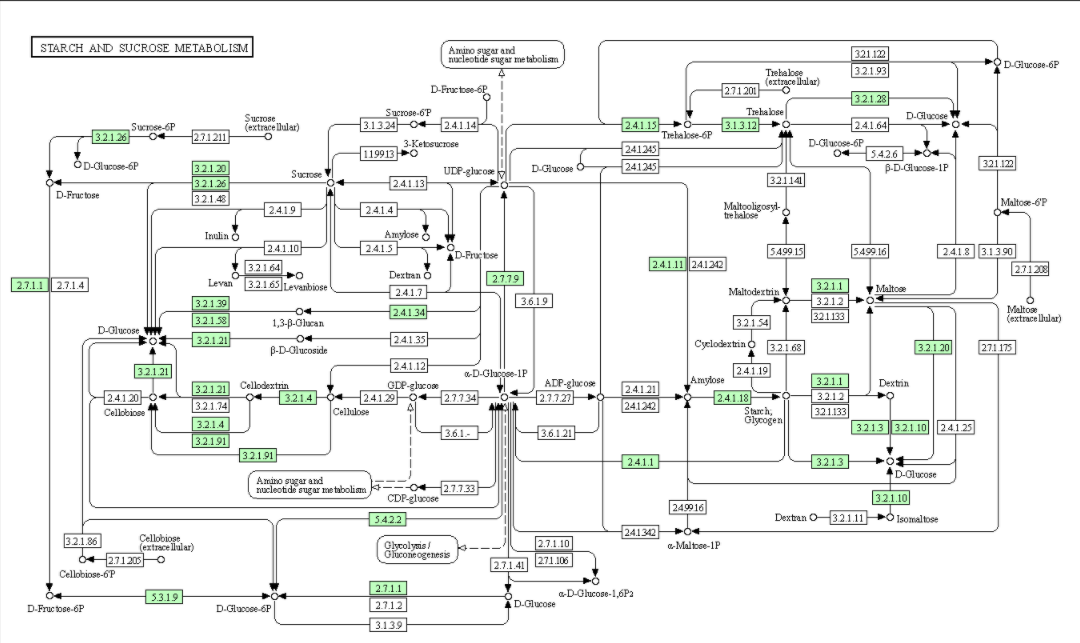


**Supplementary Fig. S3**. Mapped reference pathway of starch and sucrose metabolism in *M. oryzae* RMg_Dl.


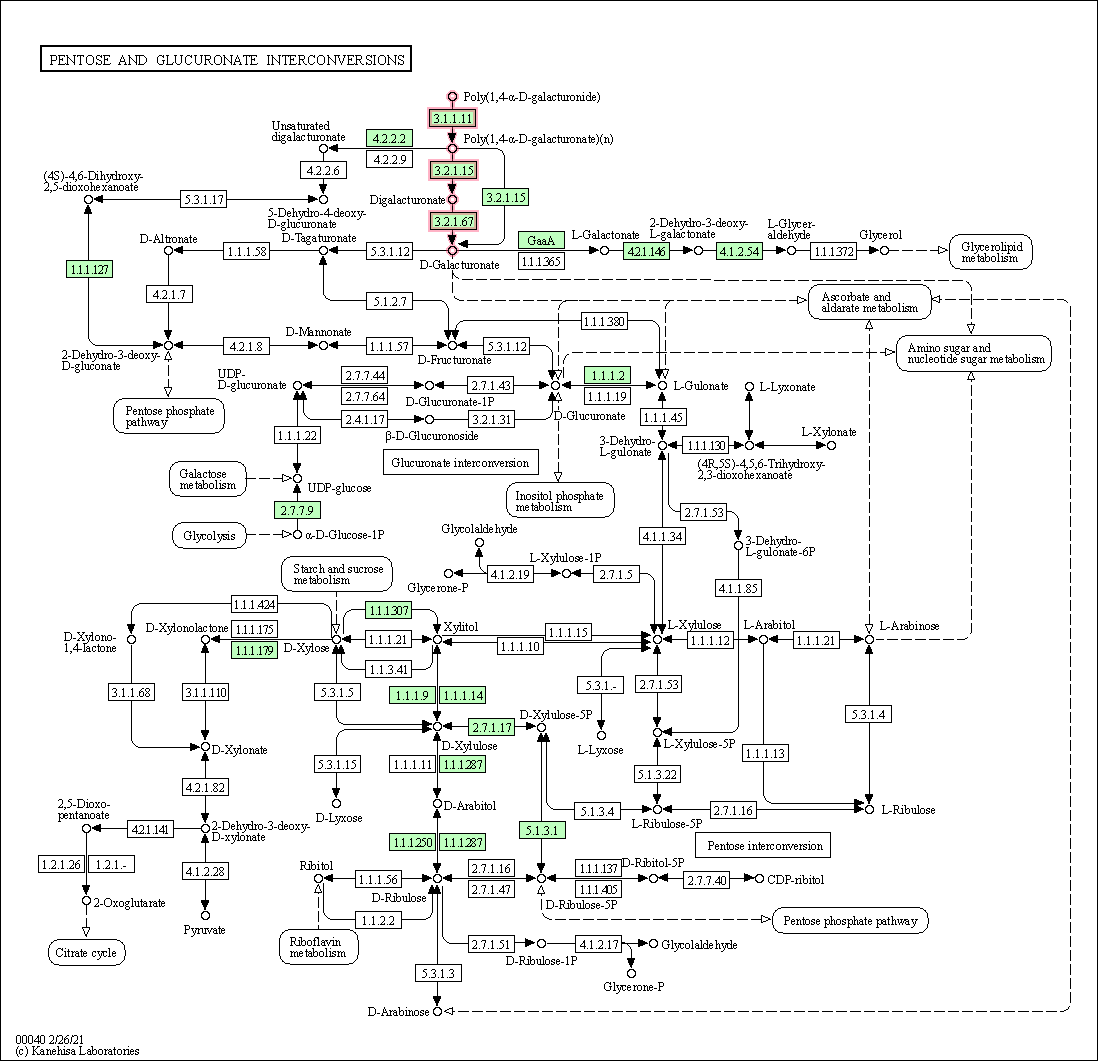


**Supplementary Fig. S4**. Mapped reference pathway of pentose and glucuronate interconversion in *M. oryzae* RMg_Dl.


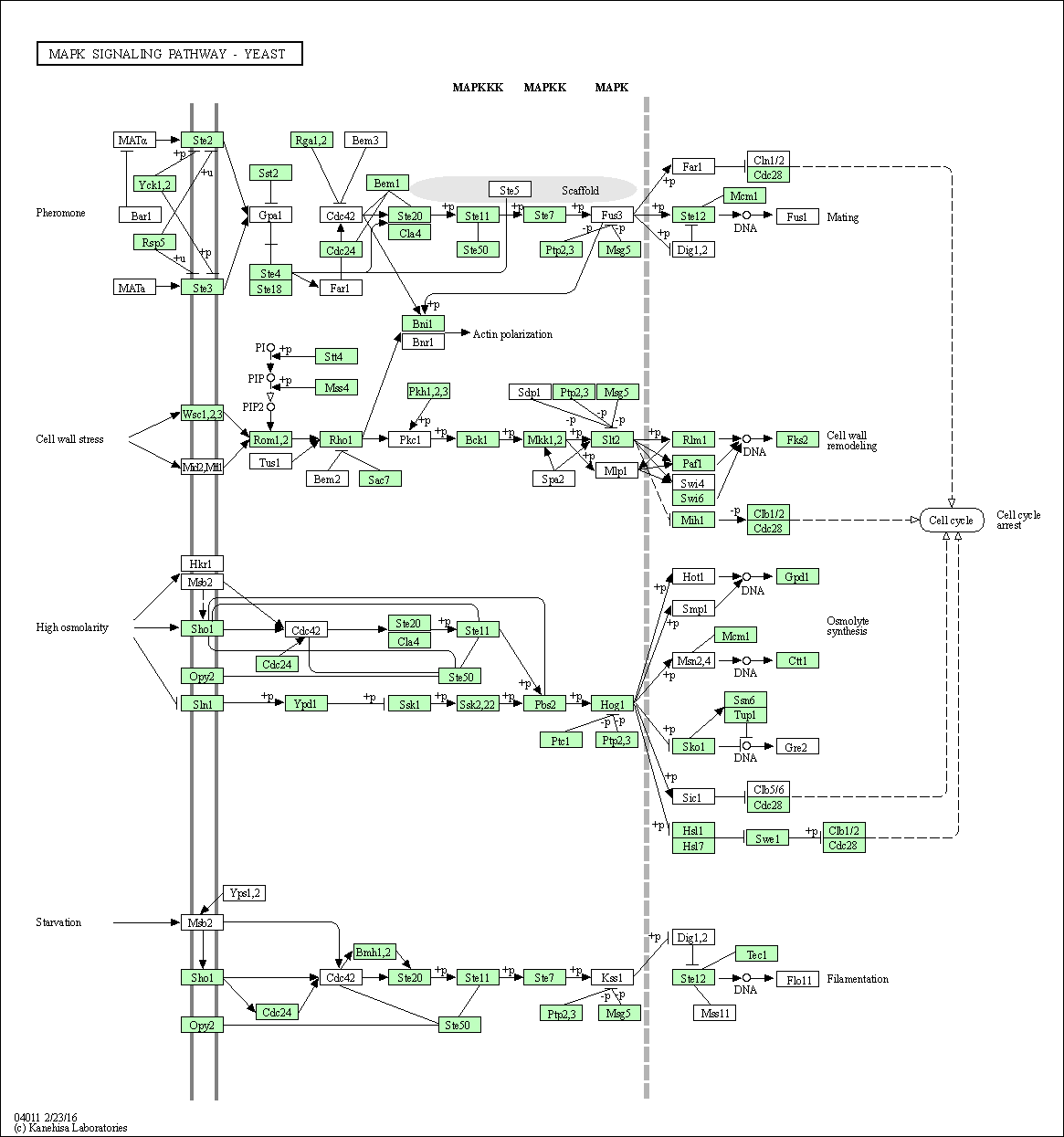


**Supplementary Fig. S5**. Mapped reference pathway of MAPK signaling pathway in *M. oryzae* RMg_Dl.

**
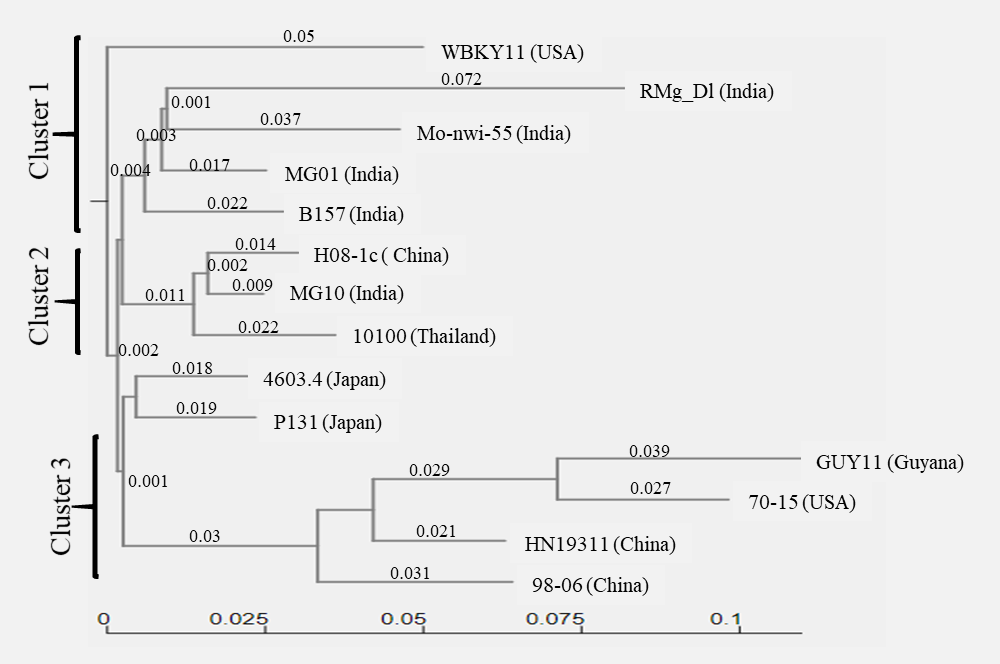
**

**Fig. S6:** The phylogenetic tree of M. oryzae genomes obtained from different country. The tree was generated using progressive alignment approach then tree was generated with Neighbour Joining method. The used genomes accession numbers are RMg_Dl (RMg_Dl; India), GCA_002924685.1 (WBKY11; USA), GCA_002021675.1 (Mo-nwi-55; India), GCA_000969745.1 (MG01; India), GCA_000832285.1 (B157; India), GCA_002218355.1 (H08-1c; China), GCA_001936435.1 (MG01; India), GCA_003991345.1 (10100; Thailand), GCA_000734215.1 (4603.4; Japan), GCA_000292605.1 (P131; Japan), GCA_002368485.1 (GUY11; Guyana), GCA_000002495.2_70-15 (70-15; USA), GCA_000475075.1 (HN19311; China), GCA_000805855.1 (98-06; China)
